# BFNet: a full-encoder skip connect way for medical image segmentation

**DOI:** 10.3389/fphys.2024.1412985

**Published:** 2024-08-02

**Authors:** Siyu Zhan, Quan Yuan, Xin Lei, Rui Huang, Lu Guo, Ke Liu, Rong Chen

**Affiliations:** ^1^ Institute of intelligent computing, University of Electronic Science and Technology of China, Chengdu, China; ^2^ Trusted Cloud Computing and Big Data Key Laboratory of Sichuan Province, Chengdu, China; ^3^ School of Computer Science and Engineering (School of Cybersecurity), University of Electronic Science and Technology of China, Chengdu, China; ^4^ School of Optoelectronic Science and Engineering, University of Electronic Science and Technology of China, Chengdu, China; ^5^ Hepatobility and Pancreatic Cen, Sichuan Provincial People’s Hospital, University of Electronic Science and Technology of China, Chengdu, China; ^6^ Department of Pulmonary and Critical Care Medicine, Sichuan Provincial People’s Hospital, University of Electronic Science and Technology of China, Chengdu, China; ^7^ Department of Cardiac Surgery, Sichuan Provincial People’s Hospital, University of Electronic Science and Technology of China, Chengdu, China; ^8^ Department of Delivery Room, Chengdu Women and Children’s Central Hospital, University of Electronic Science and Technology of China, Chengdu, China

**Keywords:** deep learning, U-net, medical image segmentation, pulmonarty embolism, CNN -convolutional neural network

## Abstract

In recent years, semantic segmentation in deep learning has been widely applied in medical image segmentation, leading to the development of numerous models. Convolutional Neural Network (CNNs) have achieved milestone achievements in medical image analysis. Particularly, deep neural networks based on U-shaped architectures and skip connections have been extensively employed in various medical image tasks. U-Net is characterized by its encoder-decoder architecture and pioneering skip connections, along with multi-scale features, has served as a fundamental network architecture for many modifications. But U-Net cannot fully utilize all the information from the encoder layer in the decoder layer. U-Net++ connects mid parameters of different dimensions through nested and dense skip connections. However, it can only alleviate the disadvantage of not being able to fully utilize the encoder information and will greatly increase the model parameters. In this paper, a novel BFNet is proposed to utilize all feature maps from the encoder at every layer of the decoder and reconnects with the current layer of the encoder. This allows the decoder to better learn the positional information of segmentation targets and improves learning of boundary information and abstract semantics in the current layer of the encoder. Our proposed method has a significant improvement in accuracy with 1.4 percent. Besides enhancing accuracy, our proposed BFNet also reduces network parameters. All the advantages we proposed are demonstrated on our dataset. We also discuss how different loss functions influence this model and some possible improvements.

## 1 Introduction

Influenced by the advancements in deep learning, computer vision techniques have been widely applied in the field of medical image analysis. Image segmentation is a critical component of medical image analysis, where accurate segmentation plays a crucial role in computer-aided diagnosis and assists doctors in lesion identification and treatment decisions. The extensive utilization of Convolutional Neural Networks (CNNs) (O’Shea and Nash, 2015) has greatly propelled the development of various segmentation models, such as FCN (Shelhamer et al., 2014), U-Net (Ronneberger et al., 2015), DeepLab (Chen et al., 2016), among others. Particularly U-Net, which stands out for its symmetric encoder-decoder and skip connections. Since its proposal in 2015, UNet has been widely adopted in medical image segmentation due to its lightweight nature and efficiency. Skip connections combine deep semantic feature maps from the decoder layers with shallow low-level feature maps from the encoder layers. Skip connections have been proven highly effective in recovering details of target objects, enabling the generation of segmentation masks (He et al., 2017) with intricate details even in complex backgrounds.

Many encoder-decoder network architectures use a series of convolutional layers and consecutive downsampling layers in the encoder to extract deep features with large receptive fields. High-resolution features from different scales of the encoder are connected via skip connections to alleviate spatial information loss caused by downsampling. Subsequently, the decoder upsamples the extracted deep features to the input resolution for pixel-level semantic prediction. Due to its simple structure and excellent performance, U-Net has achieved great success in various medical imaging applications. Following this technological path, many algorithms such as 3D U-Net (Çiçek et al., 2016), Res-UNet (Diakogiannis et al., 2019), U-Net++ (Zhou et al., 2018), and U-Net3+ (Huang et al., 2020) have emerged as image segmentation methods for various medical imaging modalities.

From the research in many segmentation papers, it is evident that feature maps at different scales contain distinct information: low-level detailed feature maps capture rich spatial information, highlighting organ boundaries, while high-level semantic feature maps contain positional information, pinpointing the location of organs. However, during upsampling and downsampling, a significant amount of information is inevitably lost, especially information related to organ boundaries.

While U-Net has made progress in providing effective feature connections, it still has some limitations. It can only access information from the current layer of the encoder, ignoring information from other layers. This results in upper-level decoders lacking access to high-level semantic feature information from lower-level encoders directly, and lower-level decoders missing low-level detailed feature information from upper-level encoders. To make decoder understanding the detailed features contained in the encoder better, U-Net++ introduces nested and dense skip connections, further strengthening the connection between the encoder and decoder. However, even with these improvements, it still cannot fully access information across all scales simultaneously. U-Net3+ ensures that each decoder layer is connected to both its encoder and the encoder of its upper layer. However, through excessively downsampling feature maps, it may lead to the loss of too much semantic information.

To address the need for more accurate segmentation in medical images and effectively reduce false positives in non-organ regions, we tried to investigate how to apply global context attention (Cao et al., 2019) to feature maps, which requiring each pixel to attend to every other pixel can be employed to solve this issue. We found that applying global context attention simply to feature maps, positioned after all encoder, mid, and decoder layers, yields optimal results since this mechanism can make the model focus more on the lesion areas. The paper which proposed global context attention also mentions that regardless of where the focus is initially placed, the model eventually attends to the corresponding regions that require attention, indicating that attention is independent of the focus point.

In summary, our main contributions are as follows: 1) We propose a novel network architecture that combines low-level detailed information with high-level semantics by introducing connections between the full-scale feature maps of the encoder and the corresponding encoder of the current layer before performing skip connections with the decoder. 2) We propose a novel connection method by fully connecting the feature maps of the encoder, enabling the decoder to learn features from allWe also noted that both upsampling and downsampling can potentially disrupt the original semantics through unnecessary generation or reduction of parameters. In the network we proposed, we have made efforts to minimize this by making minimal parameter changes to achieve the best possible results. 3) We considered a variety of models that optimize U-Net based on different objectives and combined their advantages. Then compared with the effects of using them separately, the results show that our model has achieved better results among all compared models. 4) We conduct extensive experiments on our own pulmonary embolism dataset, where BFNet consistently outperformed several baseline methods.

## 2 Related work

CNN: Early medical image segmentation methods mainly relied on traditional machine learning algorithms. The reasons for using these methods were partly due to insufficient computational power and partly due to the lack of advanced datasets and algorithms. With the development of deep CNNs, the method proposed by Ronneberger et al., in 2015 for medical image segmentation has become a standard approach in many medical image analysis tasks. Due to the simplicity and excellent performance of the U-shaped structure, various methods similar to U-Net have emerged continuously, such as nn-UNet (Isensee et al., 2018; Isensee et al., 2022), Res-UNet, Dense-Unet (Guan et al., 2018), Double U-Net (Jha et al., 2020), and U-Net3+. U-Net++ designs a series of nested and dense skip paths to reduce semantic gaps, Attention U-Net (Oktay et al., 2018) proposes a novel attention gate mechanism that allows the model to focus on targets of different shapes and sizes, UNet 3+ utilizes deep supervision and full-size skip connections, Dense-UNet leverages the advantages of U-Net’s dense and skip connections, Res-UNet adds a weighted attention mechanism. Currently, CNN-based methods have achieved tremendous success in the field of medical image segmentation, thanks to their excellent generalization ability and simple yet elegant structure.

Visual Transformer: The transformer (Vaswani et al., 2017) was first introduced for machine translation tasks. Transformer-based methods have achieved state-of-the-art performance in various tasks (Devlin et al., 2019) about natural language processing. Driven by the success of the Transformer, researchers introduced a novel Visual Transformer (ViT) (Dosovitskiy et al., 2020), which achieved promising results in image recognition tasks. SETR (Zheng et al., 2020) regards semantic segmentation as a sequence-to-sequence prediction task, using Transformer as the encoder. Subsequently, the Swin-Transformer (Liu et al., 2021), based on the shifted window mechanism, was proposed. It reduces computational complexity by introducing shift-based self-attention and has shown excellent performance in many medical segmentation tasks. Unlike most previous Transformer-based models, Swin Transformer is a hierarchical architecture that allows for flexible adjustment of the number of layers to achieve optimal performance. However, due to transformer-based models require substantial time and computational resources for training, CNNs remain active in medical image segmentation problems.

## 3 Methods

In this section, we will provide a detailed overview of the overall structure of BFNet, as illustrated in the figure. We will start by introducing U-Net, U-Net++, and U-Net3+, followed by an explanation of the specific details and improvements in our BFNet design.

### 3.1 Architecture

**FIGURE 1:**
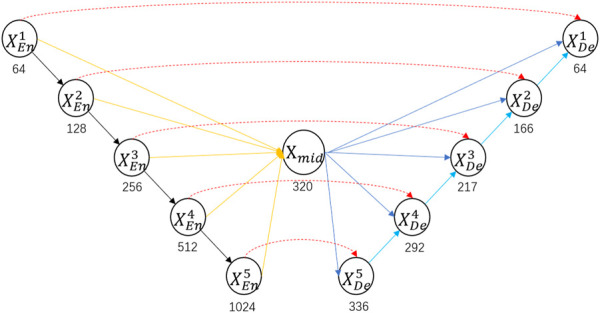
Specific architecture of BFNet. Red arrows represent skip connections between the encoder and decoder; yellow arrows represent connections synthesizing mid layers; black arrows represent downsampling; blue arrows represent skip connections between mid layer and the decoder; cyan arrows represent upsampling. Where $X_{En}$ is the encoder and $X_{De}$ is the encoder. The numbers above En and De represent the number of layers. We start from the layer closer to the original feature map.

**FIGURE 2:**
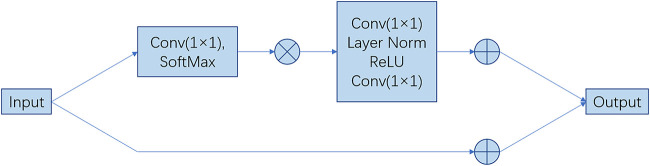
Architecture of the main blocks. The feature maps are shown as feature dimensions, where ⊗ denotes matrix multiplication, ⊕ denotes broadcast elementwise addition. The main function of the first block is to capture the global context information of the input data. It extracts global context features by modeling the global information of the input sequence. The main function of the second block is to transform and enhance the input data, combine the global context information, and generate more expressive feature representations.

In order to solve the problem that the U-Net and U-Net++ decoders cannot efficiently use encoders at different layers, we connect all the layers of the encoder and call the result the mid layer, which ensures that each layer of the decoder can obtain all the feature maps of the encoder. To simplify the image, we only show the core structure of the model in Figure 1, and we will show the specific structure in Figure 2.

### 3.2 Global context attention

Since we apply global context attention modules into each of our encoder, mid layer, and decoder, we will briefly introduce this attention mechanism.

#### 3.2.1 Non-local attention network

Instead of Non-local Attention Network (Zhang et al., 2019), Convolutional Neural Networks (CNNs) only consider pixel information within local regions when processing images, ignoring interactions between global information. Basic non-local blocks aim to strengthen features at query locations by aggregating information from other positions. Non-local blocks can be seen as global context modeling modules, aggregating specific global context features for each query position (weighted average over all positions using a query-specific attention map). It’s demonstrated that global context features modeled by non-local blocks are nearly identical at different query positions, implying that although non-local blocks aim to compute query-specific global context for each query position, the trained global context is actually independent of query positions. Therefore, it’s unnecessary to compute query-specific global context for each query position, allowing non-local blocks to be simplified and combined with Squeeze-Excitation Blocks (Hu et al., 2017) which will be mentioned later. We can learn it more in the formula 1:

$$NL_{\text{i}}=x_{\text{i}}+W_{\text{z}}\overset{H*W\left(*C\right)}{\underset{j=1}{\sum }}\frac{f\left(x_{\text{i}},x_{\text{j}}\right)}{N\left(x\right)}\left(W_{\text{v}}\times x_{\text{j}}\right)$$ (1)

where i is the index of query positions, and j enumerates all possible positions. $f\left(x_{i},x_{j}\right)$ denotes the relationship between position i and j, and has a normalization factor N(x) . $W_{z}$ and $W_{v}$ denote linear transform matrices.

#### 3.2.2 Global context network

To construct the model of global context features maps, GENet (Hu et al., 2018), and PSANet (Zhao et al., 2018) perform rescaling to different channels to recalibrate the channel dependency with global context. Global Context Network integrates the advantages of two networks. It benefits from the simplified non-local (SNL) blocks for effectively modeling long-range dependencies and also from the lightweight computation of squeeze-excitation (SE) blocks. Formula 2 is for this network, as follows:

$$GCA_{\text{i}}=x_{\text{input}}+1\times 1Conv\times \left(GlobalAttentionPooling\;x_{\text{j}}\right)$$ (2)

Therefore, it can be applied at multiple levels to better capture long-range dependencies with only a slight increase in computational cost. Its network architecture is illustrated in Figure 2.

### 3.3 Encoder

For the encoder, similar to the encoder proposed in the original U-Net, we adopted conventional convolution methods to connect each layers and learn features from the feature maps. However, our downsampling approach involves using convolution with increased stride and output channels to achieve the same downsampling effect, which is different from traditional downsampling. The purpose of increasing the stride is to reduce the resolution. Besides, simply selecting the maximum value in the spatial kernel size make the model unable to get more information from downsampling step, which may have improvements. Although this step increases computational overhead, it benefits both the mid layer and the decoder, making it worthwhile. Before convolution reduces the resolution and extracts features, we add a global context attention module at the end of each convolutional layer, so that the feature information of each layer can pay more attention to the lesion area that the model needs to pay attention. The encoder formula 3 is as follows:

$$X_{\text{EN}}^{i}=A\left(C\left(D\left(X_{\mathrm{input}}\right)\right)\right)$$ (3)

Where i indicates the number of the layer, D realizes the downsampling operation. C donates two consecutive convolutions, and A represents the global context attention mechanism.

In each layer of the encoder, we first perform two convolutions to extract the feature map information passed to the layer. Then we will use a convolution layer with a kernel size of 3×3 and a stride of 2 for downsampling, the purpose of which is to reduce the resolution while extracting useful key information to pass to the global context attention module, so that the model can recognize more important parts. Its channel attention mechanism has a compression ratio of 8 in the process of feature dimensionality reduction and dimensionality increase, which has been proven to achieve almost the best results with a small increase in parameters.

### 3.4 Mid layer

**FIGURE 3:**
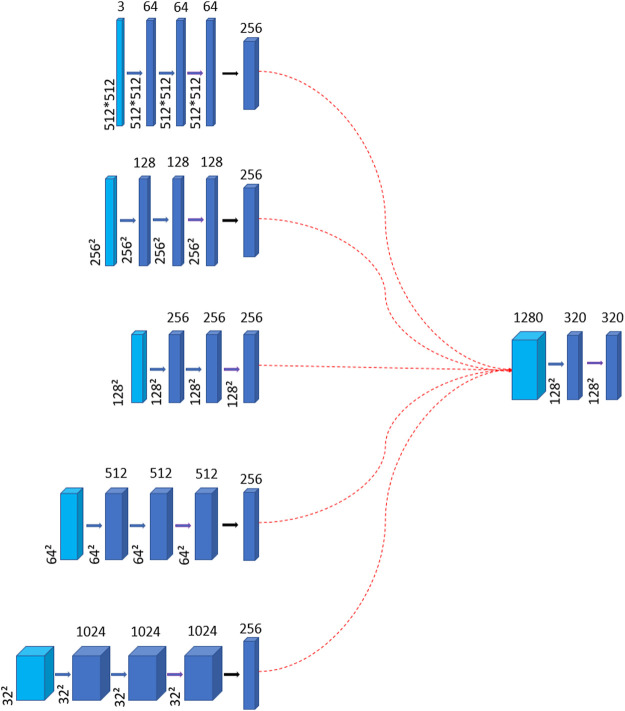
The diagram illustrates the generation of the mid layer. Thin blue arrows represent the connection formed by the layers of the encoder to create the mid layer. Thick blue arrows denote convolution operations, while thick purple arrows signify the incorporation of the global context attention module. The red dotted arrows indicate that the feature maps of all encoder layers are connected to the resulting mid layer, whose channel dimension is 1280. The intermediate layer also undergoes two convolutions to learn features. And the number of channels is reduced to 1/4 of the original, which aims to reduce the parameters.

The mid layer involves upsampling or downsampling in each layer of the encoder and uses convolutional operations to transform them into feature maps of the same size but with different channel numbers. These feature maps are then concatenated and passed through a convolutional layer to reduce their dimensionality by 1/4 of the previous, which aims to reduce the number of parameters and also to minimize the influence of noise on the feature maps. Because excessively large feature dimensions can distort the feature maps and lead to learning unnecessary noise, as shown in Figure 3. Once the feature maps are obtained, they are fed into a self-attention module to better focus on the areas indicated by the labels.

The mid layer consists of five encoder layers. Initially, we need to upsample or downsample the encoder layers to make the size of each layer is unified to 1280×128×128 , however, directly connecting them to the decoder would significantly increase the model’s parameter count. To reduce the number of parameters, we use 3×3 convolutions to reduce their channel numbers to one-fourth of the original. From Table 4, we can find that this has almost no impact on the model’s performance, while substantially reducing the parameter count.

**FIGURE 4:**
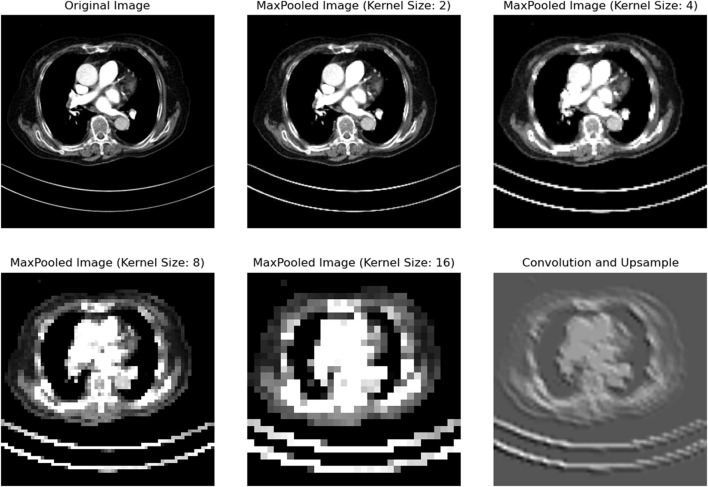
After the CT images are reduced in resolution at different magnifications, we can see that the image is still relatively clear when the resolution is reduced to 1/2 and 1/4 of the original, but after being reduced to 1/8 or smaller, obvious pixel blocks appear, indicating that the details have been lost. The last picture is restored by upsampling the high-dimensional image. It can be seen that the organ boundary and position details have been lost a lot, which is the reason why we try to avoid upsampling.

We conducted extensive research on the design of parameters in the mid layer. We applied convolutional operations to reduce a lung image to 1/2, 1/4, 1/8, and 1/16 of its original size, as shown in the figure. From Figure 4, it can be seen that downsampling by a factor of 4 or more leads to significant information loss. Therefore, we aimed to keep the downsampling scale within 1/4 as much as possible.

Downsampling simply compresses information, with all information in the feature maps originating from the real image. In contrast, the information in upsampled feature maps is inferred and generated based on existing feature maps. In designing the network, we also aimed to avoid generating feature map information through upsampling as much as possible. The more details can be known in the formulas (4–7):

$$X_{\text{mid}}^{part1}=\sum A\left(RB\left(C\left(D\left(X_{EN}^{\text{i}}\right)\right)\right)\right.\;\;\;\;\;i=1,2$$ (4)

$$X_{\text{mid}}^{part2}=A\left(RB\left(C\left(X_{EN}^{3}\right)\right)\right)$$ (5)

$$X_{\text{mid}}^{part3}=\sum A\left(RB\left(C\left(U\left(X_{EN}^{\text{j}}\right)\right)\right)\right.\;\;\;\;\;j=4,5$$ (6)

$$X_{\text{mid}}=X_{\text{mid}}^{part1}+X_{\text{mid}}^{part2}+X_{\text{mid}}^{part3}$$ (7)

Where A represents the global context attention mechanism, while RB corresponds to ReLU and BatchNorm. C indicates reducing the number of channels in the convolutional layers to one-fourth of their original size, while D signifies downsampling. U is used to denote upsampling. Part1 is the first two layers of the encoder, which need to be downsampled to reduce the resolution while increasing the feature map dimension to obtain more specific information. Part2 is the third layer of the encoder, because its resolution is already the same as the required resolution, it only needs to expand its dimensional channels. Part3 is the last two layers of the encoder, because its resolution is less than 256, it can only be restored by upsampling to reduce the number of channels.

Through the above operation, we will get a 1280∗256∗256 mid layer feature map. But if we want to connect it directly to each decoder layer, the feature map of each decoder layer will be too large. To avoid this, we use a convolution layer with a convolution kernel of 3×3 to reduce its number of channels to 1/4 of the original. We prove in the following experimental section that it has almost no effect on the model effect. Finally, we put the feature map into the global context attention module to obtain better performance.

### 3.5 Decoder

The decoder of this paper adopts a new double-jump connection method, which aims to make full use of the information exchange between the encoder and the decoder to improve the performance. First, the feature map of the mid layer that has passed the global context attention module is connected with the feature map of the encoder at the same layer to make full use of the semantic information extracted from the encoder, and combined with the local spatial information extracted from the decoder, so that to obtain a richer and more accurate feature representation. This jump connection method helps the decoder to better understand the entire input image and accurately reconstruct the target image during the decoding process. We need to use different step sizes because the resolution size to be restored is also different. The maximum step size of upsampling and downsampling is 4.

Then, the decoder feature map that has been upsampled by the 4×4 convolution kernel is connected to the previously obtained feature map as a new feature map. The advantage of this connection method is that the resolution of the feature map can be restored through the upsampling operation and fused with the previous feature map, thus effectively retaining the detailed information of the image. At the same time, since the 4×4 convolution kernel can change the resolution to an even number, it avoids the need to discard some information or add invalid data by padding when the feature map resolution is an odd number, ensuring the stability and reliability of the network.

Finally, a 1×1 convolution is used to reduce the number of channels of the concatenated feature map to a quarter of the original one. Such operations help reduce the number of parameters, reduce the computational burden of the model. At the same time, by reducing the number of channels of the feature map, some useless information can be effectively removed, and the generalization ability and performance of the model can be improved while speeding up the training speed. The formulas (8, 9) for the decoder are as follow:

$$X_{\text{DE}}^{i}=\left\{A\left(RB\left(C\left(X_{\text{DE}}^{i+1}+X_{\text{mid}}+X_{\text{EN}}^{i}\right)\right)\right)\right\}\;\;\;\;\;i=1,2,3,4$$ (8)

$$X_{\text{DE}}^{5}=\left\{A\left(RB\left(C\left(X_{\text{mid}}+X_{\text{EN}}^{5}\right)\right)\right)\right\}$$ (9)

Where A represents the global context attention mechanism, RB stands for the ReLU function and BatchNorm operation, and C denotes reducing the channel dimension. i indicates the layer number where the formula is located. Because the decoder of layer 5 does not receive any information from the decoder, it only needs to connect the encoder of this layer and the middle layer.

### 3.6 Images enhancement

Image enhancement is one of the commonly used techniques in digital image processing. The purpose of image enhancement technology is to improve the quality of the image to achieve a pleasing effect. The work usually needs to be done is to remove the noise in the image, make the edges clear, and highlight certain properties in the image.

The characteristics of medical images are often complex and variable. Therefore, the model needs to accurately identify and analyze images under different conditions. Image enhancement can help the model adapt better to various image transformations and perturbations, thus improving its generalization ability. We performed random rotation and normalization on the images, which enhances data complexity and reducing computational overhead. Additionally, we prepared for situations where medical images may vary in size. When detecting images smaller than the specified size, we resized them using the BICUBIC method to match the specified size. Conversely, if the image size exceeds the specified size, we cropped it accordingly. Although such situations did not occur in our dataset, we incorporated this design to keep the robustness of the program.

## 4 Experiments

### 4.1 Dataset

The model was trained and validated on CT images of pulmonary embolism of the liver. The dataset comes from patient data provided by Sichuan Provincial People’s Hospital. It includes 1196 processed abdominal CT scans, of which 1075 and 112 are used for training and testing respectively. In order to speed up the training, the label images have been processed in advance, and the label part is processed to RGB value 1. Dice Loss is the evaluation standard used in this paper. Its Dice score indicator can not only be used for model training, but also can be directly used as an indicator to judge the effect of the model in this dataset. The formula of Dice loss is formula 10, as follows:

$$DiceLoss=1-\frac{2\times \sum_{i}^{N}pi\times t_{i}}{\sum_{i}^{N}p_{i}^{2}+\sum_{i}^{N}t_{i}^{2}}$$ (10)

where i denotes the index of pixels, representing the position of each pixel in the image. $p_{i}$ represents the probability value of the *i*th pixel in the predicted segmentation result, while $t_{i}$ represents the label value of the *i*th pixel in the ground truth segmentation result. N represents the total number of pixels in the image.

### 4.2 Network comparison

**FIGURE 5:**
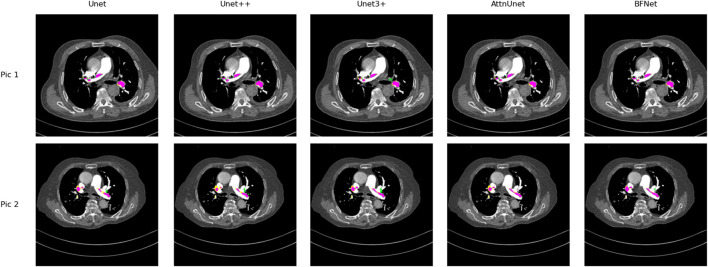
Comparison of the performance of Unet, Unet++, Unet3+, AttnUnet, and BFNet on this dataset. Purple area: True positive (TP), where samples correctly predicted as positive; Yellow area: False negative (FN), where samples actually positive are incorrectly predicted as negative; Green area: False positive (FP), where samples actually negative are incorrectly predicted as positive. It is easy to find that BFNet perform well with most of color is purple.

In this section, we compare our proposed BFNet with various network architectures. Under standard network models, BFNet achieved the best performance, with improvements of 2.1% and 1.4% over U-Net and U-Net++, respectively, on this dataset. Considering that medical images may contain lesions that are not accurately labeled, we visualize metrics such as TP and FP. As shown in Figure 5, we observe that BFNet provides more accurate delineation of boundaries and isolated regions, and it performs well in segmenting extremely small pulmonary embolisms.

### 4.3 Implementation details

BFNet is implemented using Python 3.11 and PyTorch 2.2.0. For all training cases, data augmentation techniques such as flipping and rotation were applied to increase data diversity. The input image size and patch size were set to 512×512×3 . Our model was trained on an Nvidia RTX 4090 GPU with 24 GB of memory. During training, the batch size was set to 4, the random seed was set to 2024, and the popular Adam optimizer was used with a cosine annealing strategy. The momentum was set to 0.9, the initial maximum learning rate was set to 1e-4, and the minimum learning rate was set to 1e-7.

### 4.4 Validation set

Our validation set divides all the images used for validation into groups of ten. The average is calculated for each group, and then these averages are summed up to obtain the overall average. This approach helps eliminate occasional results that may be either better or worse on certain images by chance, thus providing a more reliable assessment of performance.

### 4.5 Results

Table 1 compares the parameter count and segmentation accuracy of U-Net and U-Net++ in the pulmonary embolism segmentation task. From the table, it can be observed that U-Net++ achieves a slight improvement of 0.1% in accuracy compared to U-Net in this task, with only a marginal average increase of 0.7% in IoU. However, U-Net++ exhibits a significantly larger number of mid parameters compared to U-Net, which we consider not to signify a significant performance improvement. As for U-Net3+, it achieves an accuracy improvement of 0.3% over U-Net in this task, with a 1.1% increase in IoU. Nevertheless, it has a large number of mid parameters, requiring a higher demand on the training platform, and it is not the optimal model for our needs. Our proposed network demonstrates improvements across all metrics and performs relatively well, and it only increase acceptable size of model. The performance of modesl are as follows.

**TABLE 1:** Performance on the dataset using different network architectures, with mIOU, Dice, mPA, and mPrecision as metrics. The best results are highlighted in bold.

Architecture	Dice	mIOU	mPA	mPrecision
U-Net w/o DS	0.8817	83.41	90.53	90.44
U-Net++ w/o DS	0.8815	84.16	90.56	90.55
U-Net3+ w/o DS	0.8822	84.53	90.95	90.76
Attention Unet	0.8902	84.17	90.49	90.73
BFNet w/o DS	**0.8956**	**85.53**	**91.45**	**91.64**

### 4.6 Ablation study

To investigate the impact of different factors on model performance, we conducted an ablation study on our model. Specifically, we examined reducing the number of network layers, increasing network dimensions, removing self-attention, and incorporating attention mechanisms into U-Net to demonstrate the robustness and generalization ability of our approach.

#### 4.6.1 Effect of network Depth

We designed a network with only 3 layers and compared it with the original 4-layer network. They were identical in all aspects except for the number of network layers. After training, we observed that the 4-layer network outperformed the 3-layer network. However, the 3-layer network still achieved decent performance with only a slight sacrifice in performance. It may be particularly effective in video detection scenarios. A network with a 5-layer encoder-decoder structure was also designed to prove that 4 layers is the best structure. It can be seen that the performance difference between 4 and 5 layers is very small, but the increase in parameters is very significant, reaching almost 7 times. Specific results are presented in Table 2.

**TABLE 2:** Performance of BFNet with different numbers of layers, with the best results highlighted in bold.

Layers	Dice	mIOU	mPA	mPrecision
3	0.8914	84.68	90.81	91.11
4	0.8956	**85.53**	**91.45**	**91.64**
5	**0.8962**	85.23	91.19	91.49

#### 4.6.2 Effect of global context attention

To demonstrate the effectiveness of global context attention on segmentation, we incorporated the global context attention mechanism into both U-Net and BFNet. The results are shown in Table 3. The results confirm the necessity and effectiveness of global context attention.

**TABLE 3:** Performance of Unet and BFNet with and without attention mechanism, with the best results highlighted in bold.

Architecture	Dice	mIOU	mPA	mPrecision
U-Net w/o Attn	0.8914	83.41	90.53	90.44
BFNet w/o Attn	0.8788	84.16	90.16	91.05
U-Net with Attn	**0.9051**	84.75	90.91	91.11
BFNet with Attn	0.8956	**85.53**	**91.45**	**91.64**

#### 4.6.3 Effect of different dimensions

In our proposed model, we reduced the number of channels to one-fourth in the middle and decoder layers to validate our approach of reducing parameters. We also trained a model without reducing parameters for comparison. The results are shown in Table 4. It can be observed that while the model’s parameter count significantly decreased, there was almost no difference in performance, demonstrating that reducing parameters was a correct and effective approach.

**TABLE 4:** Performance of high-dimensional BFNet *versus* low-dimensional BFNet, with the best results highlighted in bold.

Architecture	Dice	mIOU	mPA	mPrecision
BFNet High Dim	**0.8932**	84.58	**91.46**	91.56
BFNet Low Dim	0.8856	**85.53**	91.45	**91.64**

### 4.7 Discussions

When reviewing the literature in related fields, we noticed that many network models use deep supervision and new loss calculation methods, such as U-Net3+’s deep supervision and classification-guided module (CGM). These methods have been proven to improve the training effect and performance of the model in their proposed papers. However, considering the particularity of the dataset processed by this paper and the control requirements for the number of model parameters, it was finally decided to temporarily exclude these methods from this test and adopt a simpler and more intuitive naive algorithm. This can not only see the purest comparison of model performance, but also build confidence for researchers who later use the model proposed in this paper as a baseline model to add other methods to the model.

In future research, we plan to add new loss calculation methods and deep supervision mechanisms to the proposed network in order to improve the training effect of the model. Although this may lead to an increase in the number of model parameters, in practice, more sophisticated loss calculation methods and supervision mechanisms often lead to better training results, thereby improving the performance of the model on the task.

After trying multiple loss functions, we found that there are some difficulties in superimposing multiple loss functions, and it is difficult to determine the proportional relationship between the loss functions, and the effect is not satisfactory. Therefore, it was finally decided to use only a single standard loss function in the model of this paper. The binary cross-entropy loss function cannot directly provide a value that can be used as a model evaluation criterion like the Dice loss, even though its effect is basically the same as the Dice loss function and has better performance. Therefore, the loss function used in all models of this paper is the Dice loss function.

## 5 Conclusion

In this paper, we propose a novel network architecture called BFNet, which utilizes full-scale connections between encoder layers, enabling precise segmentation of pulmonary embolisms. We use Dice Loss as our evaluation metric, as it is crucial for assessing model performance and effectively reduces the impact of false positives. Experimental results on our pulmonary embolism dataset demonstrate that BFNet outperforms many state-of-the-art and classical approaches, achieving the goal of highly accurate segmentation.

## Data Availability

The raw data supporting the conclusions of this article will be made available by the authors, without undue reservation.
